# Temporal Dynamics of Epigenetic Aging and Frailty From Midlife to Old Age

**DOI:** 10.1093/gerona/glad251

**Published:** 2023-10-27

**Authors:** Jonathan K L Mak, Ida K Karlsson, Bowen Tang, Yunzhang Wang, Nancy L Pedersen, Sara Hägg, Juulia Jylhävä, Chandra A Reynolds

**Affiliations:** Department of Medical Epidemiology and Biostatistics, Karolinska Institutet, Stockholm, Sweden; Department of Medical Epidemiology and Biostatistics, Karolinska Institutet, Stockholm, Sweden; Department of Medical Epidemiology and Biostatistics, Karolinska Institutet, Stockholm, Sweden; Department of Medical Epidemiology and Biostatistics, Karolinska Institutet, Stockholm, Sweden; Department of Clinical Sciences, Danderyd Hospital, Karolinska Institutet, Stockholm, Sweden; Department of Medical Epidemiology and Biostatistics, Karolinska Institutet, Stockholm, Sweden; Department of Medical Epidemiology and Biostatistics, Karolinska Institutet, Stockholm, Sweden; Department of Medical Epidemiology and Biostatistics, Karolinska Institutet, Stockholm, Sweden; Faculty of Social Sciences (Health Sciences) and Gerontology Research Center (GEREC), University of Tampere, Tampere, Finland; Department of Psychology, University of California, Riverside, California, USA; Institute for Behavioral Genetics, University of Colorado Boulder, Boulder, Colorado, USA; (Medical Sciences Section)

**Keywords:** DNA methylation, Dual change score models, Epigenetic clock, Frailty, Longitudinal

## Abstract

**Background:**

DNA methylation-derived epigenetic clocks and frailty are well-established biological age measures capturing different aging processes. However, whether they are dynamically linked to each other across chronological age remains poorly understood.

**Methods:**

This analysis included 1 309 repeated measurements in 524 individuals aged 50–90 years from the Swedish Adoption/Twin Study of Aging. Frailty was measured using a validated 42-item frailty index (FI). Five epigenetic clocks were calculated, including 4 principal component (PC)-based clocks trained on chronological age (PCHorvathAge and PCHannumAge) and aging-related physiological conditions (PCPhenoAge and PCGrimAge), and a pace of aging clock (DunedinPACE). Using dual change score models, we examined the dynamic, bidirectional associations between each of the epigenetic clocks and the FI over age to test for potential causal associations.

**Results:**

The FI exhibited a nonlinear, accelerated increase across the older adulthood, whereas the epigenetic clocks mostly increased linearly with age. For PCHorvathAge, PCHannumAge, PCPhenoAge, and PCGrimAge, their associations with the FI were primarily due to correlated levels at age 50 but with no evidence of a dynamic longitudinal association. In contrast, we observed a unidirectional association between DunedinPACE and the FI, where a higher DunedinPACE predicted a subsequent increase in the FI, but not vice versa.

**Conclusions:**

Our results highlight a temporal order between epigenetic aging and frailty such that changes in DunedinPACE precede changes in the FI. This potentially suggests that the pace of aging clock can be used as an early marker of the overall physiological decline at system level.

Aging is associated with complex, dynamic interactions of ­biological processes at multiple hierarchical scales from molecular to organismal level, which subsequently leads to cumulative decline in physiological systems, increased vulnerability to diseases, and mortality (1,2). To evaluate interventions that target aging, several measures of biological aging, including DNA methylation-derived epigenetic clocks and frailty, have therefore been developed to quantify various aging processes (2–4). Epigenetic clocks are algorithms that combine methylation levels at specific cytosine-phosphate-guanine (CpG) sites in the genome to predict age and aging-related phenotypes. First-generation clocks, such as HorvathAge (5) and HannumAge (6), were trained to predict chronological age. Second-generation clocks, such as PhenoAge (7) and GrimAge (8), additionally include clinical information to predict morbidity and mortality. DunedinPACE is a third-generation clock that was trained on longitudinal changes in biomarkers and estimates the pace of aging (9). Different from epigenetic clocks, which are generally considered as measures of molecular or cellular aging (4), frailty is a measure of systemic aging that reflects a state of increased vulnerability to stressors due to multisystem decline (10,11). Among the multiple operational definitions, the frailty index (FI) is most commonly used to capture the accumulation of age-related deficits at organ and system levels (11,12).

Although both epigenetic clocks and frailty have consistently been shown to predict age-related diseases and mortality (13,14), there has been inconclusive evidence on whether they may be linked to each other apart from their mutual correlation with chronological age. Although cross-sectional studies mostly suggested significant associations between frailty and the first- and second-generation epigenetic clocks (15,16), some longitudinal studies (17,18), but not all (19), reported null associations between epigenetic clocks measured at the study baseline and subsequent changes in frailty. Moreover, these studies usually had relatively short follow-up time, and the bidirectional relationships between epigenetic clocks and frailty, including the recently developed pace of aging clock, have not been examined. The “geroscience hypothesis” posits that age-related diseases, including frailty and multimorbidity, are consequences of aging at the molecular/cellular level, and by targeting the underlying molecular pathways these diseases could be prevented or postponed (20). Since epigenetic clocks and frailty capture age-related changes at different hierarchical scales (3,4), understanding their dynamic interplay across age, that is, whether changes in epigenetic clocks temporally precede changes in frailty, or vice versa, can shed light into how multiple aging mechanisms interact with each other over time (20). Moreover, as the current knowledge on epigenetic clocks is mainly based on cross-sectional data (21), a better understanding of how different generations of epigenetic clocks change longitudinally across age is crucial for their future translation into clinical trials and practice, which could ultimately help to improve the aging processes (2).

To this end, we applied bivariate dual change score models (DCSMs) in a sample of Swedish twins who were followed up to 20 years to explore the dynamic associations between 5 epigenetic clocks and frailty from midlife through late-life.

## Method

### Study Population

We used data from the Swedish Adoption/Twin Study of Aging (SATSA) (22), which was drawn from the population-based Swedish Twin Registry (23). SATSA is a longitudinal study of same-sex twins consisting of up to 10 in-person testing (IPT) waves performed at approximately 3-year intervals from 1984 to 2014. Each IPT included a health examination and blood sample collection. For this analysis, we included only data from the third, fifth, sixth, eighth, ninth, and tenth IPT waves when DNA methylation data were available. A total of 524 individuals who participated in at least one IPT and had information on both frailty and DNA methylation were included.

This study was approved by the Regional Ethics Review Board in Stockholm (Dnr 2016/1888-31/1). Informed consent was obtained from all participants.

### Epigenetic Clocks

Whole blood DNA methylation levels were measured using Illumina’s Infinium Human Methylation 450K or MethylationEPIC BeadChip, and the raw data were preprocessed using a rigorous quality control pipeline as detailed elsewhere (24). DNA methylation-based epigenetic clocks, including HorvathAge, HannumAge, PhenoAge, and GrimAge have previously been derived in SATSA using methylation data in 353, 71, 513, and 1030 CpGs, respectively (4). However, recent studies suggested that these clocks can be rather unreliable due to technical noise from the individual CpGs, especially in longitudinal settings (25). Hence, we used the proposed principal component (PC) versions of epigenetic clocks in this analysis, including PCHorvathAge, PCHannumAge, PCPhenoAge, and PCGrimAge, which were trained from the PCs of CpGs that capture the majority of the age-related signals and have shown to be more reliable for studying longitudinal trajectories (25). We also included DunedinPACE as a measure of the pace of aging, which was trained on longitudinal data capturing within-individual decline in 19 biomarkers of organ-system integrity (representing cardiovascular, metabolic, renal, hepatic, immune, dental, and pulmonary systems) in individuals of the same chronological age (9). As its construction already excluded the unreliable CpGs, a PC training is not needed for the DunedinPACE (9). The 4 PC-clocks were measured in years, whereas DunedinPACE was measured in biological year per chronological year (a value of 1 can be interpreted as a rate of 1 year of biological aging per year of chronological aging (9)).

### Frailty Index

Frailty was measured using a 42-item FI, which was developed and validated in SATSA using deficit items from self-reported diseases, signs, symptoms, and activities of daily living (Supplementary Table 1) (26). In accordance with the deficit accumulation model (12), the FI at each wave was calculated as the sum of the deficits divided by the total number of items considered, yielding a score of 0–100%, where a higher score represents a higher degree of frailty.

### Statistical Analysis

Pearson’s correlations between the epigenetic clocks and the FI at baseline were calculated. We used DCSMs to study changes in the epigenetic clocks and FI and with age independently in univariate models and jointly in bivariate models (27). For modeling purposes, we split the data into 2-year age intervals from 50 to <52 years through 88 to <90 years (data from 90 years onwards were sparse and therefore excluded from the analysis). In all models, chronological age (in 2-year bins) was adjusted for as the underlying timescale, and sex was adjusted for by regressing it on the intercepts and slopes.

**Figure 1.**
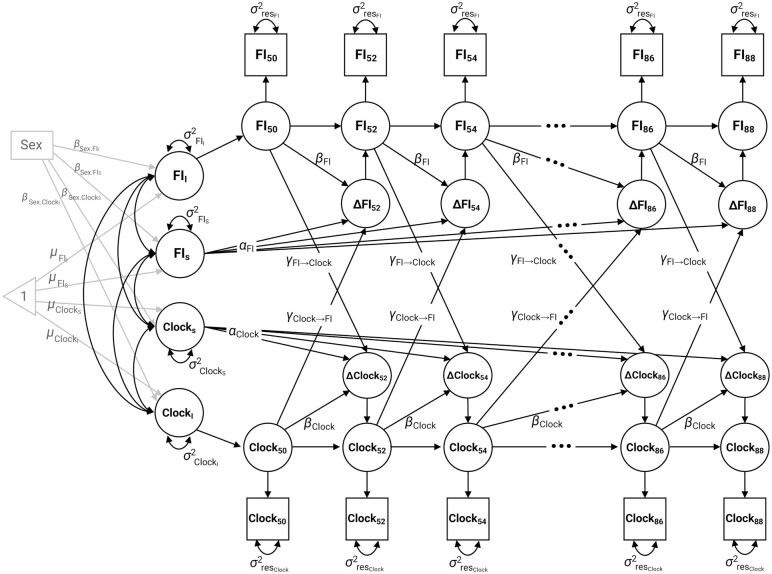
Path diagram of the bivariate dual change score model in assessing the relationship between age changes in the frailty index (FI) and epigenetic clocks. “Clock” denotes each of the 5 DNA methylation-derived epigenetic clocks: PCHorvathAge, PCHannumAge, PCPhenoAge, PCGrimAge, and DunedinPACE. The FI and epigenetic clocks are modeled in 2-year age intervals from 50 to <52 years (FI_50_, Clock_50_) through 88 to <90 years (FI_88_, Clock_88_). FI_I_ and Clock_I_ represent intercepts (at age 50) and FI_S_ and Clock_S_ represent their linear slopes. 𝜇_FI_ and 𝜇_Clock_ represent the estimated mean levels of the intercepts and slopes, and σ^2^_FI_ and σ^2^_Clock_ represent individual variations around the mean intercepts and slopes. α_FI_ and α_Clock_ represent constant change related to the slope factors FI_S_ and Clock_S_, which are fixed to 1 in the model. β_FI_ and β_Clock_ represent the proportional (nonlinear) change effects in FI and epigenetic clocks (β_Clock_ was not included in the models for PCHorvathAge, PCHannumAge, and DunedinPACE due to nonsignificant proportional effect estimates), which are relative to the level at the previous occasion. γ_FI→Clock_ and γ_Clock→FI_ represent the cross-trait coupling effects, where FI at the previous occasion can influence change in the clocks, and vice versa. Sex is added as a covariate in the model, where β_Sex.FI_ and β_Sex.Clock_ represent the regression coefficients of sex on the intercepts and slopes. All unlabeled single-headed arrows are set to 1. The unlabeled 2-headed arrows in the left represent the covariances between the intercepts and slopes. All the systematic variance and covariance are also estimated on the twin-pair level to account for twin relatedness. Residual variance (σ^2^_res_, indicating variation not accounted for by the model) is assumed to be constant within each trait at each age. Residual covariance between the FI and epigenetic clocks is also estimated but not shown in the figure.

A series of univariate DCSMs was first fitted to characterize the longitudinal trajectory of each measure (path diagram of the model is shown in the upper and lower parts of Figure 1). Similar to latent growth curve models, the univariate DCSMs consist of an intercept and a slope for assessing the mean trajectory and individual differences around change in each measure over age, thus capturing both within-person and between-person differences in change. Two components of change were incorporated in the model: a static linear change component, which is defined as α (normally set to 1) multiplied by the slope factor (FI_S_ and Clock_S_), and a proportional change component (β_FI_ and β_Clock_) that depends on the previous score. For example, the equation for change in the FI at age *t*, without considering epigenetic clocks, can be written as ΔFI_*t*_ = α × FI_S_ + β_FI_ × FI_*t−*1_. The model also estimates the means, variances, and covariances of the intercept and slope, as well as the residual variance. We calculated the variances and covariances for the intercept and linear slope at both individual- and twin pair-levels to account for twin relatedness. To test whether there is evidence of a nonlinear change for the FI and epigenetic clocks over age, we compared the goodness of fit of the full univariate models specified above with models removing the proportional change parameter (ie, leaving only the static linear parameter α × FI_S_ or α × Clock_S_).

Based on the best-fitting univariate models, we then fitted bivariate DCSMs to assess dynamic associations between the epigenetic clocks and FI. On top of the univariate models, the bivariate DCSMs additionally include cross-trait covariances of the intercepts and slopes to measure static associations, as well as 2 coupling parameters from FI to epigenetic clock and vice versa (γ_FI→Clock_ and γ_Clock→FI_) to test for dynamic lead-lag relationships (Figure 1). The equation for change in the FI at age *t* in relation to previous levels of epigenetic clock can then be written as ΔFI_*t*_ = α × FI_S_ + β_FI_ × FI_*t*−1_ + γ_Clock→FI_ × Clock_*t*−1_. Same as the proportional change component, the coupling parameters depend on the score at the previous occasion and as such represent a dynamic relationship on top of the static correlations between intercepts and slopes. By comparing models with and without the coupling parameters, we can therefore test whether there is evidence of a temporal, dynamic association between the 2 processes above and beyond the static associations. Evidence of such coupling effects is in line with, but not proof of a causal relationship. We first compared a full-coupling (bidirectional) model to a no-coupling model removing both coupling parameters. The full-coupling model was further compared to 2 models including each of the coupling parameters to test for unidirectional associations.

All analyses were performed in R v.4.2.3, and the DCSMs were fitted using full-information maximum likelihood estimation in *OpenMx* (version 2.20.6). Comparisons of the goodness of fit of models were done by likelihood ratio tests, where a *p* value < .05 comparing a constrained model with a full model indicates a significant loss in model fit.

## Results

### Sample Characteristics

The study sample included 1 309 repeated measures in 524 individuals, the mean age of which was 68.2 years (*SD* 9.2) at baseline and 58.6% were women (Table 1). Participants were followed up to 6 waves spanning a maximum of 20 years. At baseline, all the epigenetic clocks were modestly correlated with the FI (*r*s ranged from 0.21 to 0.35); these correlations were largely attenuated after adjusted for chronological age, with the strongest correlation observed for DunedinPACE and FI (*r* = 0.16; Supplementary Figure 1).

**Table 1.** Baseline Characteristics of the Included SATSA Participants

Characteristic	*N* or Mean
No. of individuals	524
No. of observations	1 309
No. of available measurements per person	
One	157
Two	123
Three	113
Four	89
Five	41
Six	1
Age, y, mean (*SD*)	68.2 (9.2)
Women, *n* (%)	307 (58.6)
Zygosity	
Monozygotic	181 (34.5)
Dizygotic	342 (65.3)
Unknown	1 (0.2)
FI (%), mean (*SD*)	9.77 (8.20)
PCHorvathAge, y, mean (*SD*)	59.98 (8.79)
PCHannumAge, y, mean (*SD*)	63.32 (8.53)
PCPhenoAge, y, mean (*SD*)	61.60 (8.60)
PCGrimAge, y, mean (*SD*)	76.74 (7.07)
DunedinPACE, mean (*SD*)	1.05 (0.15)

*Note*: FI = frailty index; SATSA = Swedish Adoption/Twin Study of Aging; *SD =* standard deviation.

### Univariate Trajectories of Epigenetic Clocks and Frailty Index

**Figure 2.**
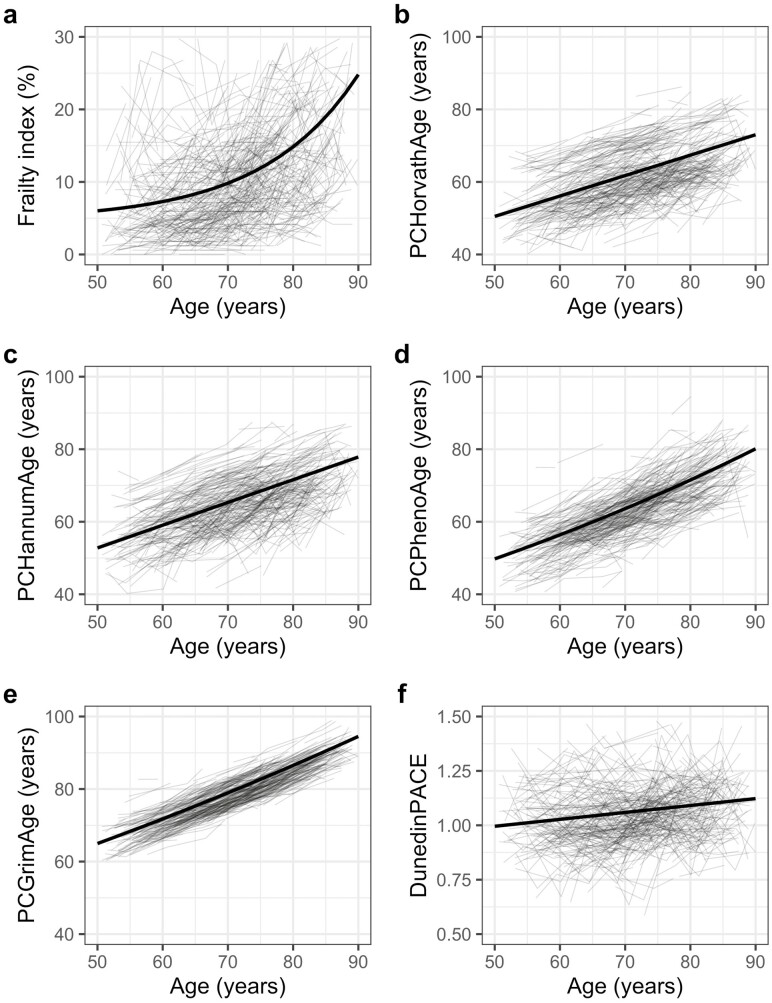
Trajectories of the frailty index and the 5 epigenetic clocks in SATSA (*n* = 524). (a) Frailty index; (b) PCHorvathAge; (c) PCHannumAge; (d) PCPhenoAge; (e) PCGrimAge; and (f) DunedinPACE. The thick black lines represent the estimated trajectories from the best-fitting univariate dual change score models of each trait. SATSA, Swedish Adoption/Twin Study of Aging.

We first fitted univariate DCSMs to examine the trajectories of the FI and epigenetic clocks. As shown in Supplementary Table 2, removing the proportional change parameter resulted in a significantly reduced fit for the univariate models of the FI, PCPhenoAge, and PCGrimAge (all *p* < .01), but not for the models of PCHorvathAge, PCHannumAge, and DunedinPACE (*p* > .05). This suggested evidence of nonlinearity in the longitudinal changes in the FI, PCPhenoAge, and PCGrimAge, although a nonlinear change was most evident for the FI, which showed an accelerated growth after age 75 years (Figure 2). For instance, the FI, PCPhenoAge, and PCGrimAge on average increased 1.3%, 6.6 years, and 6.8 years between ages 50 and 60, and increased 10.0%, 8.6 years, and 8.0 years between ages 80 and 90, respectively. Meanwhile, PCHorvathAge, PCHannumAge, and DunedinPACE had a steady 10-year increase of 5.6 years, 6.3 years, and 0.03 across all ages, respectively (Figure 2). These patterns can similarly be seen from the parameter estimates of the best-fitting models (Supplementary Table 3); the mean FI level at age 50 was 6.01%, with an overall negative linear slope of −0.69 but a positive proportional effect (β_FI_ = 0.15) that drives up the FI across age. In contrast, there was a positive mean slope for all the epigenetic clocks, and a positive proportional effect for PCPhenoAge and PCGrimAge, so that the clocks generally increased linearly with age (Figure 2).

### Longitudinal Associations Between Epigenetic Clocks and Frailty Index

Bivariate DCSMs were then fitted to examine dynamic associations between epigenetic clocks and FI over age. For the bivariate models between the 4 PC-clocks and the FI, removing both coupling parameters did not lead to a significant loss in model fit, indicating no evidence of a dynamic association after accounting for the static correlations between intercepts and slopes (ie, neither the PC-clocks nor the FI predict subsequent changes of the other; Supplementary Table 4). Despite lack of a coupling effect, we observed positive correlations between intercepts of the PC-clocks and the FI (eg, covariance between intercepts of PCGrimAge and FI = 3.46), thus suggesting a between-person, static association between PC-clocks and the FI such that their values were positively correlated at age 50 (Table 2). This is also consistent with the positive Pearson’s correlations observed between the clocks and the FI at baseline (Supplementary Figure 1). Moreover, individuals with higher PCHannum and PCPhenoAge at age 50 tended to have a significantly slower growth in the FI, as shown by the negative covariances between slope of the clocks and intercept of the FI, which were −1.24 and −1.32, respectively (Table 2).

**Table 2.** Estimates From the Best-Fitting Bivariate Dual Change Score Models Between the Frailty Index and Each of the Five Epigenetic Clocks

	PCHorvathAge	PCHannumAge	PCPhenoAge	PCGrimAge	DunedinPACE
	Estimate (*SE*)	Estimate (*SE*)	Estimate (*SE*)	Estimate (*SE*)	Estimate (*SE*)
Best-fitting model	No coupling	No coupling	No coupling	No coupling	DunedinPACE to FI
FI parameters					
Mean intercept at age 50	6.03 (0.51)*	6.08 (0.51)*	6.04 (0.51)*	6.07 (0.50)*	6.55 (0.58)*
Mean slope	−0.73 (0.23)*	−0.77 (0.23)*	−0.73 (0.23)*	−0.77 (0.23)*	−12.31 (4.35)*
Proportional change effect (β_FI_)	0.15 (0.02)*	0.16 (0.02)*	0.15 (0.02)*	0.16 (0.02)*	0.06 (0.03)
Effect of sex on intercept (women vs men)	2.39 (0.79)*	2.40 (0.79)*	2.46 (0.78)*	2.50 (0.77)*	0.60 (1.10)
Effect of sex on slope (women vs men)	−0.36 (0.15)*	−0.37 (0.15)*	−0.37 (0.15)*	−0.39 (0.15)*	0.29 (0.28)
Variance of intercept, individual level	19.95 (5.05)*	20.40 (4.93)*	20.58 (5.09)*	19.82 (4.78)*	9.94 (5.10)
Variance of slope, individual level	0.52 (0.19)*	0.56 (0.20)*	0.54 (0.19)*	0.54 (0.19)*	1.10 (0.56)
Covariance of intercept and slope, individual level	−3.15 (0.96)*	−3.31 (0.96)*	−3.26 (0.95)*	−3.22 (0.92)*	−2.24 (1.06)*
Variance of intercept, twin pair level	6.71 (4.48)	6.63 (4.30)	6.04 (4.65)	6.54 (4.33)	2.66 (4.16)
Variance of slope, twin pair level	0.12 (0.15)	0.12 (0.15)	0.10 (0.15)	0.12 (0.15)	0.71 (0.60)
Covariance of intercept and slope, twin pair level	−0.91 (0.81)	−0.92 (0.80)	−0.79 (0.84)	−0.90 (0.81)	0.29 (0.92)
Residual variance	15.52 (0.94)*	15.48 (0.93)*	15.48 (0.93)*	15.65 (0.94)*	14.52 (0.95)*
Epigenetic age parameters					
Mean intercept at age 50	50.49 (0.61)*	52.77 (0.60)*	49.79 (0.54)*	65.00 (0.29)*	10.04 (0.13)*
Mean slope	1.12 (0.04)*	1.25 (0.04)*	0.37 (0.38)	0.58 (0.24)*	0.06 (0.01)*
Proportional change effect (β_Clock_)	—	—	0.02 (0.01)*	0.01 (0.00)*	—
Effect of sex on intercept (women vs men)	−2.43 (1.23)	−2.07 (1.21)	−1.03 (0.87)	−2.90 (0.47)*	0.01 (0.22)
Effect of sex on slope (women vs men)	0.05 (0.07)	0.00 (0.08)	−0.04 (0.07)	0.03 (0.03)	−0.03 (0.02)*
Variance of intercept, individual level	5.89 (3.65)	4.53 (4.01)	9.67 (3.60)*	4.45 (1.04)*	1.01 (0.30)*
Variance of slope, individual level	0.06 (0.04)	0.02 (0.04)	0.06 (0.04)	0.01 (0.01)	0.00 (0.00)*
Covariance of intercept and slope, individual level	−0.26 (0.37)	0.07 (0.42)	−0.35 (0.36)	−0.20 (0.09)*	−0.05 (0.02)*
Variance of intercept, twin pair level	54.16 (8.44)*	43.53 (8.15)*	18.13 (4.51)*	5.29 (1.27)*	0.56 (0.11)*
Variance of slope, twin pair level	0.00 (0.03)	0.03 (0.04)	0.02 (0.03)	0.01 (0.01)	—
Covariance of intercept and slope, twin pair level	−0.61 (0.45)	−0.70 (0.49)	−0.58 (0.36)	−0.15 (0.09)	—
Residual variance	9.85 (0.58)*	12.24 (0.70)*	6.21 (0.37)*	1.60 (0.10)*	0.99 (0.05)*
Bivariate parameters					
Coupling effect, clock to FI (γ_Clock→FI_)	—	—	—	—	1.19 (0.43)*
Coupling effect, FI to clock (γ_FI→Clock_)	—	—	—	—	—
Covariance intercept FI—intercept clock, individual level	6.27 (3.19)*	6.89 (3.42)*	8.00 (3.54)*	3.46 (1.63)*	1.94 (1.15)
Covariance slope FI—intercept clock, individual level	−1.06 (0.57)	−1.24 (0.63)*	−1.32 (0.63)*	−0.60 (0.31)	−0.96 (0.26)*
Covariance intercept FI—slope clock, individual level	−0.47 (0.33)	−0.47 (0.35)	−0.55 (0.35)	−0.22 (0.14)	−0.13 (0.12)
Covariance slope FI—slope clock, individual level	0.08 (0.06)	0.09 (0.06)	0.10 (0.06)	0.04 (0.03)	0.04 (0.02)*
Covariance intercept FI—intercept clock, twin pair level	−2.41 (3.42)	−4.37 (4.54)	−0.50 (4.30)	−0.61 (1.53)	−0.17 (0.54)
Covariance slope FI—intercept clock, twin pair level	0.65 (0.59)	1.04 (0.79)	0.26 (0.75)	0.22 (0.27)	−0.64 (0.29)*
Covariance intercept FI—slope clock, twin pair level	0.12 (0.26)	0.26 (0.32)	0.03 (0.34)	0.02 (0.12)	—
Covariance slope FI—slope clock, twin pair level	−0.03 (0.04)	−0.06 (0.06)	−0.02 (0.06)	−0.01 (0.02)	—
Residual covariance between FI and clock	−0.05 (0.53)	−0.01 (0.58)	0.30 (0.41)	0.20 (0.21)	0.03 (0.15)

*Notes*: FI = frailty index; *SE =* standard error.

Model fit statistics are shown in Supplementary Table 4. DunedinPACE used in the models was multiplied by 10 to ease calculation. For the DunedinPACE models, only variance of the intercept was calculated at twin pair level due to convergence issues. *p < .05.

**Figure 3.**
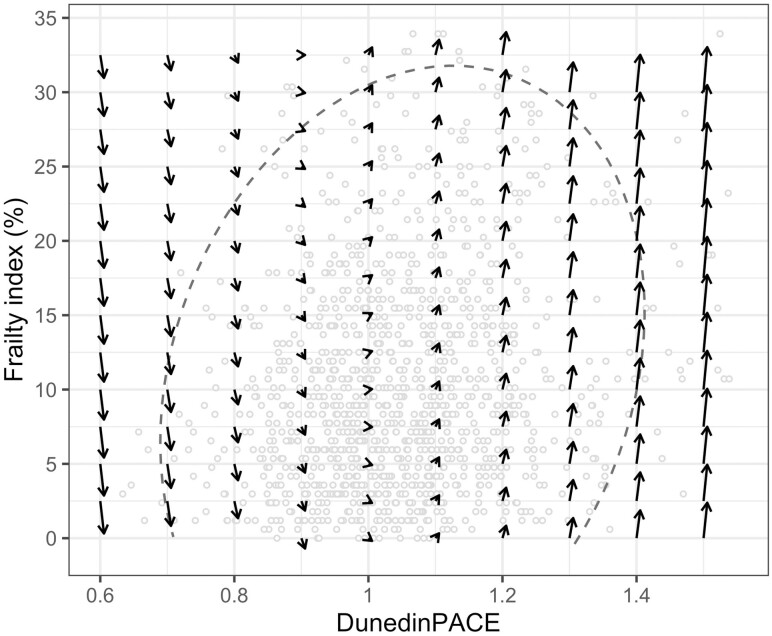
Vector field plot visualizing the dynamic relationship between DunedinPACE and frailty index. The vector field is constructed based on the bivariate dual change score model for frailty index and DunedinPACE. The values for DunedinPACE were in the original scale, where a value of 1 is interpreted as a rate of 1 year of biological aging per year of chronological aging. Each arrow in the plot represents the direction and relative magnitude of the expected changes in both the frailty index (y-axis) and DunedinPACE (x-axis) for every given pair of values. The dashed line represents the 95% ellipse, and the dots represent the actual data points (*n* = 524).

On the other hand, there was a significant coupling effect from DunedinPACE to FI (γ_Clock→FI_ = 1.19), but not from FI to DunedinPACE, indicating a unidirectional association such that DunedinPACE was a positive leading indicator of subsequent changes in the FI (Supplementary Table 4; Table 2). In addition, compared to the univariate model of the FI, the negative linear slope was stronger (FI_S_ = −12.31) and the positive proportional effect attenuated (β_FI_ = 0.06) in the bivariate model (Table 2). Together with the coupling parameter, this indicates that changes in DunedinPACE have substantial effects on subsequent estimates of the FI, where a low DunedinPACE predicts a stable or even decreasing FI, whereas a high DunedinPACE predicts a substantial increase in the FI. To visualize the dynamic relationship between DunedinPACE and the FI over time, we presented a vector field plot in Figure 3, which is usually used as a graphical display of coupled dynamical systems (28). Each arrow in the plot represents the expected direction and relative magnitude of changes in DunedinPACE and FI for each combination of their initial values. The plot also displays the actual data points and a 95% ellipse; the focus should be on the arrows contained within the ellipse (28). Along the horizontal lines (eg, when FI equals 15%), as DunedinPACE increases, the arrows point further upwards, indicating a greater increase in the FI (ie, a positive coupling from DunedinPACE to changes in FI). On the contrary, as FI increases along the vertical lines, there is no obvious change in the length of arrows in the horizontal direction, indicating no significant coupling from FI to changes in DunedinPACE.

## Discussion

By applying DCSMs in a longitudinal twin study of aging, this study assessed the longitudinal trajectories of frailty and 5 epigenetic clocks and examined the direction of their interrelationships across the older adulthood. In particular, we demonstrated a unidirectional, dynamic association between the pace of aging clock and the FI, where a higher level in DunedinPACE temporally preceded increase in the FI. However, the first- and second-generation epigenetic clocks (ie, PCHorvathAge, PCHannumAge, PCPhenoAge, and PCGrimAge) did not appear to be dynamically coupled with the FI over age, although we did observe static associations as indicated by their correlated levels at age 50.

From the univariate DCSMs, we observed an accelerated increase for the FI, PCPhenoAge, and PCGrimAge across age, but not for the PCHorvathAge, PCHannumAge, and DunedinPACE. The PCHorvathAge (5) and PCHannumAge (6) are first-generation clocks designed for chronological age prediction, thus inherently implying a linear progression with age. On the other hand, the second-generation clocks (PCPhenoAge and PCGrimAge) (7,8) and the FI (11) are measures for predicting mortality risk, and the DunedinPACE is a measure of the *rate* of aging where a linear growth would be indicative of an accelerated change (9). Therefore, these findings potentially suggest that the rate of aging becomes faster as individuals approach the later stages of life.

Few studies have assessed the longitudinal associations between epigenetic clocks and frailty, and most of them were limited to the use of first- and second-generation clocks that were trained on cross-sectional measures of age and aging-related traits. Using data from the Canadian Longitudinal Study on Aging, Verschoor et al. showed that HannumAge and GrimAge were associated with a small increase in the FI over 3 years of follow-up (19). Contrarily, Seligman et al. found only correlations between the FI and HannumAge, PhenoAge, and GrimAge at baseline of the MOBILIZE Boston cohort, but none of these clocks were associated with changes in the FI over 18 months (18). Other studies similarly reported absence of a longitudinal association between epigenetic clocks and markers of physical frailty (17,29). In the present study, using bivariate DCSMs, we only observed positive correlations between levels of the PC-clocks and FI at age 50 years and negative correlations between levels of PCHannumAge and PCPhenoAge at age 50 and the FI slope, but no other intercept-slope correlations or dynamic coupling effect over age. Taken together, the current evidence does not support for a temporal, causal connection between the first- and second-generation clocks and frailty. Thus, any dynamics may occur at earlier ages than examined here, an unknown or unmodeled factor may contribute to the associations between the PC-clocks and FI, or the PC-clocks may be less sensitive indices of change.

Meanwhile, we found a significant coupling effect from DunedinPACE, a pace of aging clock, to the FI. Different from the earlier versions of epigenetic clocks, DunedinPACE is a novel measure of biological aging reflecting the ongoing rate of deterioration in system integrity (9). As such, DunedinPACE may be more sensitive to capture age-related changes at the molecular level and more indicative of an acceleration in the underlying aging processes compared to other clocks. Many of the biomarkers used in construction of the DunedinPACE such as glycated hemoglobin, cholesterols, and C-reactive protein have also been associated with frailty (30). The observed unidirectional, dynamic relationship may therefore imply a temporal order in the aging process, where a higher pace of aging measured by molecular or epigenetic markers could lead to a subsequent increase in deficit accumulation at organ or system levels. Alternatively, other factors such as genes affecting biological aging may first lead to changes on the molecular and cellular levels, before their effects manifest on tissue and whole organismal level. These results also provide support for the geroscience hypothesis, suggesting that slowing down the aging processes at the molecular or cellular level may delay or prevent many age-related diseases and frailty (2,20). Additionally, these results strengthen the potential use of DunedinPACE as an early marker to monitor the overall physiological decline during aging, although it would be important for future studies to replicate our results and assess if DunedinPACE may be associated with physical frailty and other age-related diseases.

Strengths of this study include the long follow-up and multiple testing occasions per person, allowing us to apply the powerful DCSMs to study dynamic interactions between frailty and epigenetic clocks over age. Instead of the traditional epigenetic clocks calculated on individual CpGs, we included the newer versions of PC-based clocks, which are more reliable in studying longitudinal changes (25). Nevertheless, some limitations should also be considered. Given the relatively small sample size and limited statistical power, we were unable to perform further analysis, such as studying sex-specific effects or investigating the impacts of other potential confounders. Although the longitudinal nature of the data allowed us to examine potential causal connections between epigenetic clocks and the FI, our observational results do not provide proof of causal relationships. As in other longitudinal studies, sample attrition over time could have resulted in a more selected sample of healthy individuals, although the full-information maximum likelihood estimation is beneficial in handling missing data due to attrition. Finally, as our sample included only older Swedish twin individuals, more studies are needed to test whether our findings are generalizable to other populations and ethnic groups.

In conclusion, we did not find support for a dynamic relationship between the first- and second-generation epigenetic clocks and frailty beyond their correlated levels at age 50. However, for DunedinPACE that is trained on changes in biomarkers and reflects the pace of aging, it is unidirectionally linked to frailty such that within-person changes in DunedinPACE temporally precede changes in the FI. These findings provide new insights into the nature of the relationships between epigenetic aging and frailty and potentially indicate a temporal, hierarchical nature of aging such that molecular changes occur prior to physiological decline at the organismal level.

## Supplementary Material

glad251_suppl_Supplementary_Tables_S1-S4_Figures_S1

## Data Availability

Methylation data are available in EMBL-EBI under accession number S-BSST1206 (https://www.ebi.ac.uk/biostudies/studies/S-BSST1206), whereas phenotypic data are available in the National Archive of Computerized Data on Aging under accession number ICPSR 3843 (https://www.icpsr.umich.edu/web/NACDA/studies/3843). Codes used for data analysis are provided at the Open Science Framework platform (https://osf.io/6cyde/?view_only=5ac1866f01a24294901e85f623288a2e).
