# Supplementary material for: Temporal Dynamics of Epigenetic Aging and Frailty From Midlife to Old Age
Source: J Gerontol A Biol Sci Med Sci. 2023 Oct 27;79(10):glad251. doi: 10.1093/gerona/glad251 (PMC11421301; doi:10.1093/gerona/glad251)
Supplement: glad251_suppl_Supplementary_Tables_S1-S4_Figures_S1 [file glad251_suppl_supplementary_tables_s1-s4_figures_s1.docx]

**Temporal dynamics of epigenetic aging and frailty from midlife to old age**

Jonathan K. L. Mak^1^*, Ida K. Karlsson^1^, Bowen Tang^1^, Yunzhang Wang^1,2^, Nancy L. Pedersen^1^, Sara Hägg^1^, Juulia Jylhävä^1,3^, Chandra A. Reynolds^4,5^

1. Department of Medical Epidemiology and Biostatistics, Karolinska Institutet, Stockholm, Sweden
2. Department of Clinical Sciences, Danderyd Hospital, Karolinska Institutet, Stockholm, Sweden
3. Faculty of Social Sciences (Health Sciences) and Gerontology Research Center (GEREC), University of Tampere, Tampere, Finland
4. Department of Psychology, University of California, Riverside, CA, USA
5. Institute for Behavioral Genetics, University of Colorado Boulder, CO, USA

*Corresponding author: Jonathan K. L. Mak

Department of Medical Epidemiology and Biostatistics, Karolinska Institutet, Nobels väg 12A, 171 77 Stockholm, Sweden

Email: jonathan.mak@ki.se

Supplemental Material

[Supplementary Figure 1. Baseline correlations of the (A) six biological age measures and (B) biological age residuals and chronological age 2](#_Toc146618441)

[Supplementary Table 1. Deficit items included in the frailty index in SATSA 3](#_Toc146618442)

[Supplementary Table 2. Model fit statistics of univariate dual change score models 4](#_Toc146618443)

[Supplementary Table 3. Parameter estimates from the best-fitting univariate models. 5](#_Toc146618444)

[Supplementary Table 4. Model fit statistics of bivariate dual change score models 6](#_Toc146618445)

| **A** | 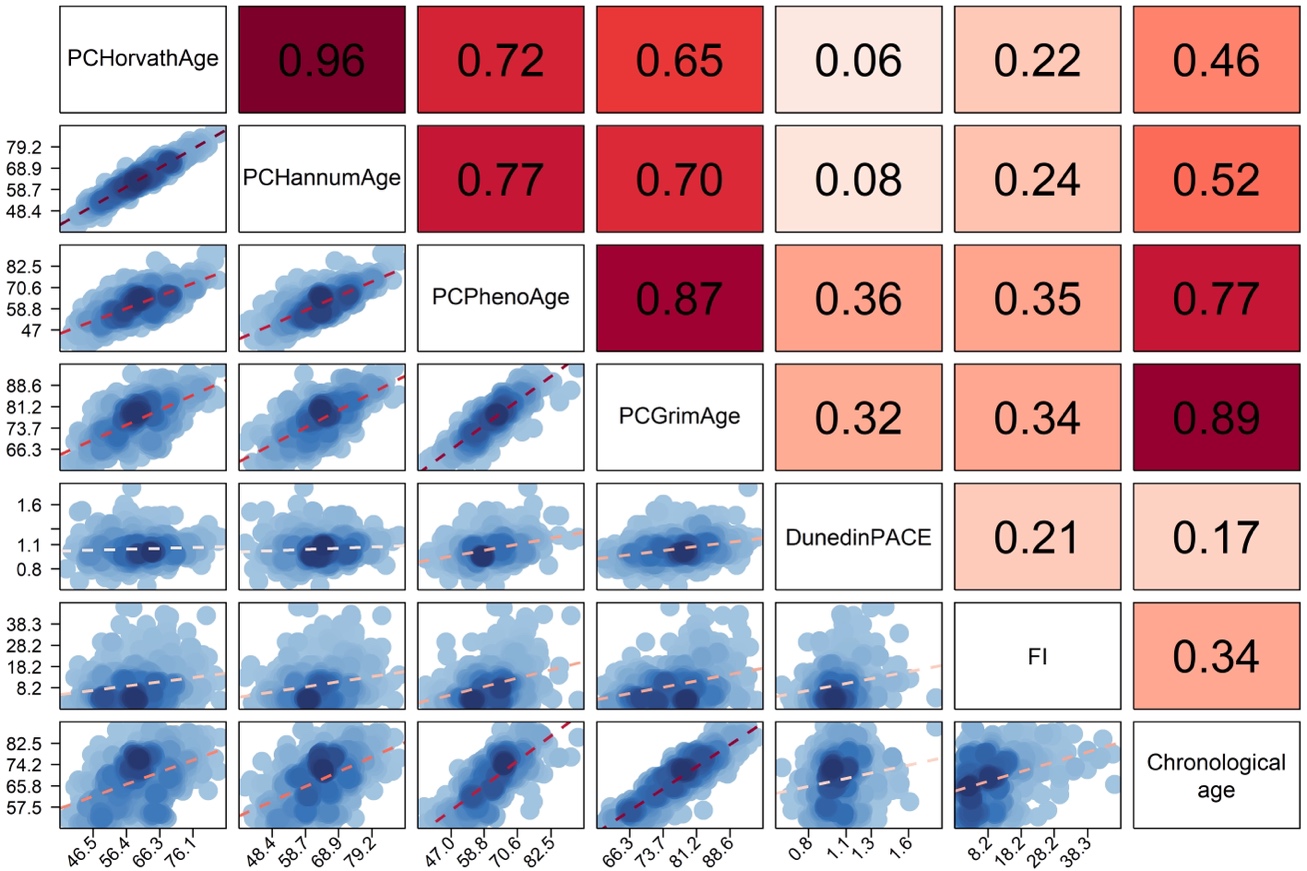 |
| --- | --- |
| **B** | 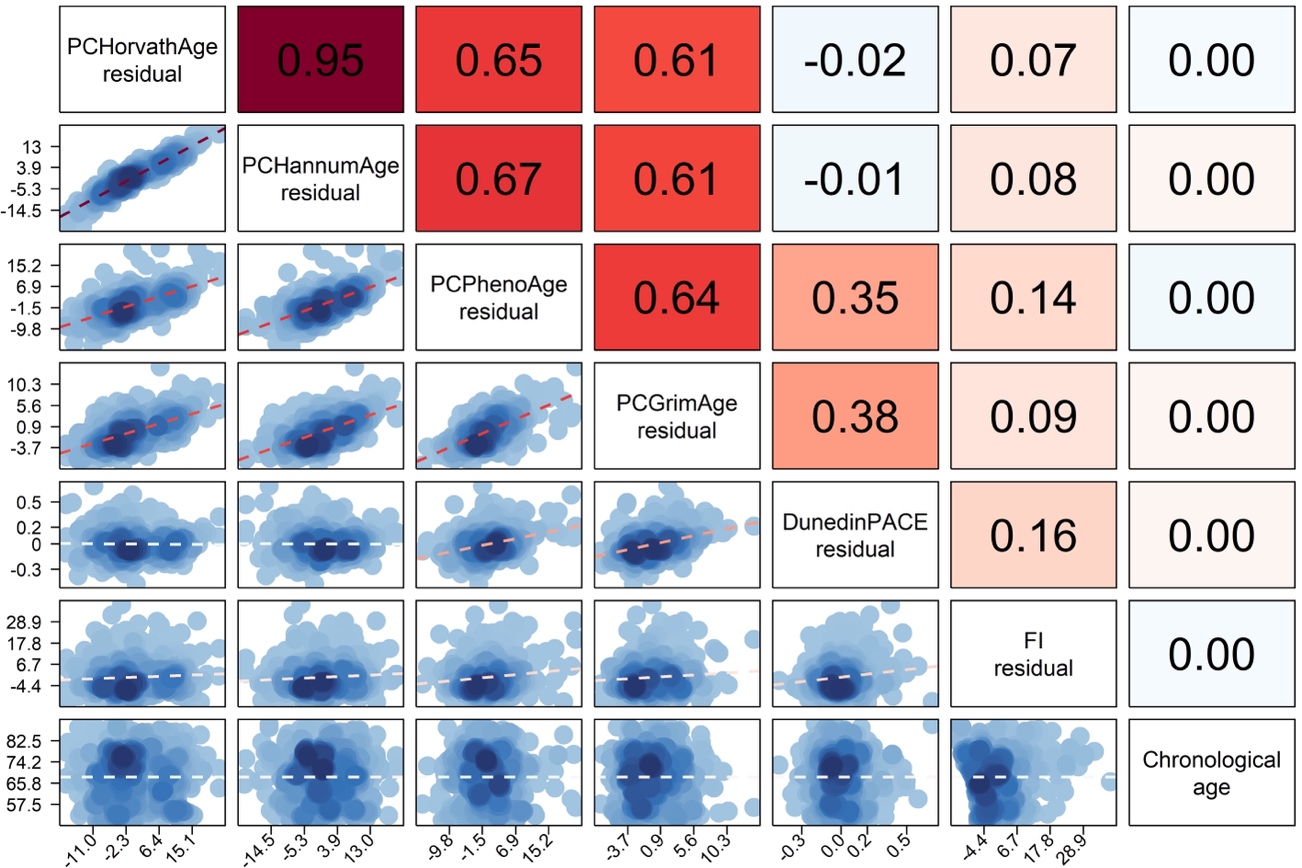 |

# **Supplementary Figure 1.** Baseline correlations of the (**A**) six biological age measures and (**B**) biological age residuals and chronological age.

Baseline was considered as the first available measurement per person. The six biological age measures include PCHorvathAge, PCHannumAge, PCPhenoAge, PCGrimAge, DunedinPACE, and frailty index (FI). Biological age residuals were calculated by regressing each biological age measure on chronological age (as a 3-degrees-of-freedom natural spline). The Pearson’s correlation coefficients (*r*) are shown in the top right triangles, and scatter plots are shown in the bottom left triangles.

# **Supplementary Table 1.** Deficit items included in the frailty index in SATSA

| No. | Item | Coding |
| --- | --- | --- |
| 1 | Allergies/allergic manifestations | No=0, Yes=1 |
| 2 | Anemia | No=0, Yes=1 |
| 3 | Arthritis | No=0, Yes=1 |
| 4 | Asthma | No=0, Yes=1 |
| 5 | Brittle bones | No=0, Yes=1 |
| 6 | Cancer or leukemia | No=0, Yes=1 |
| 7 | Cataracts | No=0, Yes=1 |
| 8 | Cerebral hemorrhage or blood clot in brain | No=0, Yes=1 |
| 9 | Chest pain | No=0, Yes=1 |
| 10 | Chronic bronchitis or emphysema | No=0, Yes=1 |
| 11 | Circulation problems in arms or legs | No=0, Yes=1 |
| 12 | Consider oneself happy and carefree | No=1, Yes=0 |
| 13 | Diabetes | No=0, Yes=1 |
| 14 | Dizziness | No=0, Yes=1 |
| 15 | Dress and undress | No problem=0, Needs help=0.5, Cannot=1 |
| 16 | Feeling depressed | Never, almost never or rather seldom=0, Quite often, always, almost always=1 |
| 17 | Feeling lonely | Never, almost never, rather seldom=0, Quite often, always, almost always=1 |
| 18 | Gastric ulcer | No=0, Yes=1 |
| 19 | Get in and out of bed | No problem=0, Needs help=0.5, Cannot=1 |
| 20 | Goiter or other gland problems | No=0, Yes=1 |
| 21 | Grocery shopping | Can shop=0, Needs help=0.5, Doesn’t shop=1 |
| 22 | Health prevents from doing things normally would like to do | No=0, Somewhat=0.5, Yes=1 |
| 23 | Hearing status | Perfect=0, Good=0.25, Pretty Good=0.5, Bad=0.75, Deaf or almost deaf=1 |
| 24 | Heart failure | No=0, Yes=1 |
| 25 | Housework | No problems=0, Needs help=0.5, Doesn’t do=1 |
| 26 | Hypertension | No=0, Yes=1 |
| 27 | Kidney disease | No=0, Yes=1 |
| 28 | Manage medications | No problems=0, Needs help=0.5, Doesn’t do=1 |
| 29 | Manage money | No problems=0, Needs help=0.5, Doesn’t do=1 |
| 30 | Persistent cough | No=0, Yes=1 |
| 31 | Prepare meals | Can plan/prepare=0, Can heat up=0.5, Doesn’t cook=1 |
| 32 | Rheumatoid arthritis | No=0, Yes=1 |
| 33 | Sciatica | No=0, Yes=1 |
| 34 | Self-grooming | No problem=0, Needs help=0.5, Cannot=1 |
| 35 | Self-reported general health | Good=0, Mediocre=0.5, Bad=1 |
| 36 | Shower and bathe | No problem=0, Needs help=0.5, Cannot=1 |
| 37 | Travel further distances | Can travel alone=0, Can go by taxi=0.5, Needs helper, special assistance or doesn’t travel=1 |
| 38 | Trouble getting to toilet in time | No=0, Yes=1 |
| 39 | Use telephone | Can look up numbers and dial=0, Needs help or doesn’t use phone=1 |
| 40 | Usually feels tired | No=0, Yes=1 |
| 41 | Vision status | Perfect=0, Good=0.25, Pretty Good=0.5, Bad=0.75, Blind or almost blind=1 |
| 42 | Walking | No problem=0, Needs help=0.5, Cannot=1 |

# **Supplementary Table 2.** Model fit statistics of univariate dual change score models

| **Model** | **AIC** | **-2LL** | **Δdf** | ***p*** |
| --- | --- | --- | --- | --- |
| FI |  |  |  |  |
| **M1: Full model** | **9198.2** | **9170.2** | **-** | **-** |
| M2: Constant change model (drop *β*) | 9256.3 | 9230.3 | 1 | <.001 |
| PCHorvathAge |  |  |  |  |
| M1: Full model | 8609.2 | 8581.2 | - | - |
| **M2: Constant change model (drop *β)*** | **8607.2** | **8581.2** | **1** | **0.86** |
| PCHannumAge |  |  |  |  |
| M1: Full model | 8737.6 | 8709.6 | - | - |
| **M2: Constant change model (drop *β*)** | **8736.0** | **8710.0** | **1** | **0.50** |
| PCPhenoAge |  |  |  |  |
| **M1: Full model** | **8126.0** | **8098.0** | **-** | **-** |
| M2: Constant change model (drop *β*) | 8132.9 | 8106.9 | 1 | 0.003 |
| PCGrimAge |  |  |  |  |
| **M1: Full model** | **6322.1** | **6294.1** | **-** | **-** |
| M2: Constant change model (drop *β*) | 6332.0 | 6306.0 | 1 | 0.001 |
| DunedinPACE |  |  |  |  |
| M1: Full model | 5132.4 | 5108.4 | - | - |
| **M2: Constant change model (drop *β*)** | **5130.9** | **5108.9** | **1** | **0.46** |

*AIC*, Akaike information criterion; *df*, degrees of freedom; *LL*, log-likelihood. *P*-values are obtained from likelihood ratio tests comparing each constrained model to the full model; a *p*-value <.05 indicates a significant loss in model fit. Constant change models are those with the proportional change parameter (*β*) removed from the full dual change score model. The best-fitting models are shown in bold.

# **Supplementary Table 3.** Parameter estimates from the best-fitting univariate models.

|  | **FI** | **PCHorvathAge** | **PCHannumAge** | **PCPhenoAge** | **PCGrimAge** | **DunedinPACE** |
| --- | --- | --- | --- | --- | --- | --- |
|  | Estimate (SE) | Estimate (SE) | Estimate (SE) | Estimate (SE) | Estimate (SE) | Estimate (SE) |
| Mean intercept at age 50 | 6.01 (0.51)* | 50.50 (0.61)* | 52.78 (0.59)* | 49.76 (0.54)* | 64.97 (0.29)* | 9.96 (0.12)* |
| Mean slope | -0.69 (0.22)* | 1.13 (0.04)* | 1.25 (0.04)* | 0.40 (0.38) | 0.62 (0.24)* | 0.06 (0.01)* |
| Proportional change effect (*β*) | 0.15 (0.02)* | - | - | 0.02 (0.01)* | 0.01 (0.00)* | - |
| Effect of sex on intercept (women vs. men) | 2.41 (0.79)* | -2.41 (1.22)* | -1.98 (1.17) | -1.02 (0.86) | -2.89 (0.47)* | -0.18 (0.25) |
| Effect of sex on slope (women vs. men) | -0.35 (0.15)* | 0.05 (0.07) | -0.01 (0.08) | -0.04 (0.07) | 0.03 (0.03) | -0.02 (0.02) |
| Variance of intercept, individual level | 20.60 (5.07)* | 6.26 (3.83) | 4.53 (3.91) | 9.44 (3.57)* | 4.35 (1.05)* | 0.92 (0.38)* |
| Variance of slope, individual level | 0.52 (0.18)* | 0.06 (0.04) | 0.02 (0.04) | 0.06 (0.04) | 0.01 (0.01) | 0.00 (0.00) |
| Covariance of intercept and slope, individual level | -3.19 (0.94)* | -0.28 (0.38) | 0.07 (0.41) | -0.33 (0.36) | -0.19 (0.09)* | -0.04 (0.03) |
| Variance of intercept, twin pair level | 6.41 (4.40) | 53.63 (8.47)* | 42.55 (7.93)* | 18.03 (4.37)* | 5.28 (1.28)* | 0.55 (0.11)* |
| Variance of slope, twin pair level | 0.10 (0.14) | 0.00 (0.03) | 0.02 (0.04) | 0.02 (0.03) | 0.01 (0.01) | - |
| Covariance of intercept and slope, twin pair level | -0.82 (0.78) | -0.59 (0.45) | -0.64 (0.47) | -0.56 (0.35) | -0.15 (0.09) | - |
| Residual variance | 15.40 (0.93)* | 9.81 (0.58)* | 12.25 (0.70)* | 6.21 (0.37)* | 1.61 (0.10)* | 0.99 (0.06)* |

*FI*, frailty index; *SE*, standard error. Model fit statistics are shown in **Supplementary Table 2**. DunedinPACE used in the models was multiplied by 10 to ease calculation. For the DunedinPACE models, only variance of the intercept was calculated at twin pair level due to convergence issues.

**p*<.05

# **Supplementary Table 4.** Model fit statistics of bivariate dual change score models

| **Model** | **AIC** | **-2LL** | **Δdf** | ***p*** |
| --- | --- | --- | --- | --- |
| PCHorvathAge & FI |  |  |  |  |
| M1: Full coupling | 17061.6 | 16989.6 | - | - |
| **M2: No coupling** | **17060.5** | **16992.5** | **2** | **0.23** |
| M3: Unidirectional, PCHorvathAge → FI | 17061.7 | 16991.7 | 1 | 0.14 |
| M4: Unidirectional, FI → PCHorvath | 17062.5 | 16992.5 | 1 | 0.09 |
| PCHannumAge & FI |  |  |  |  |
| M1: Full coupling | 17187.5 | 17115.5 | - | - |
| **M2: No coupling** | **17185.9** | **17117.9** | **2** | **0.31** |
| M3: Unidirectional, PCHannumAge → FI | 17185.6 | 17115.6 | 1 | 0.77 |
| M4: Unidirectional, FI → PCHannumAge | 17187.9 | 17117.9 | 1 | 0.12 |
| PCPhenoAge & FI |  |  |  |  |
| M1: Full coupling | 16556.8 | 16482.8 | - | - |
| **M2: No coupling** | **16555.1** | **16485.1** | **2** | **0.31** |
| M3: Unidirectional, PCPhenoAge → FI | 16555.4 | 16483.4 | 1 | 0.43 |
| M4: Unidirectional, FI → PCPhenoAge | 16556.4 | 16484.4 | 1 | 0.21 |
| PCGrimAge & FI |  |  |  |  |
| M1: Full coupling | 14754.2 | 14680.2 | - | - |
| **M2: No coupling** | **14753.9** | **14683.9** | **2** | **0.16** |
| M3: Unidirectional, PCGrimAge → FI | 14752.2 | 14680.2 | 1 | 0.84 |
| M4: Unidirectional, FI → PCGrimAge | 14755.8 | 14683.8 | 1 | 0.06 |
| DunedinPACE & FI |  |  |  |  |
| M1: Full coupling | 13543.6 | 13479.6 | - | - |
| M2: No coupling | 13552.1 | 13492.1 | 2 | 0.002 |
| **M3: Unidirectional,** **DunedinPACE → FI** | **13543.6** | **13481.6** | **1** | **0.15** |
| M4: Unidirectional, FI → DunedinPACE | 13552.4 | 13490.4 | 1 | 0.001 |

*AIC*, Akaike information criterion; *df*, degrees of freedom; *LL*, log-likelihood. *P*-values are obtained from likelihood ratio tests comparing each constrained model to the full coupling (bidirectional) model; a *p*-value <.05 indicates a significant loss in model fit. The best-fitting models are shown in bold.
